# Supplementary figures and images for: Development and In-Field Validation of an Autonomous Soil Mechanical Resistance Sensor
Source: Sensors (Basel). 2025 Mar 19;25(6):1919. doi: 10.3390/s25061919 (PMC11946251; doi:10.3390/s25061919)

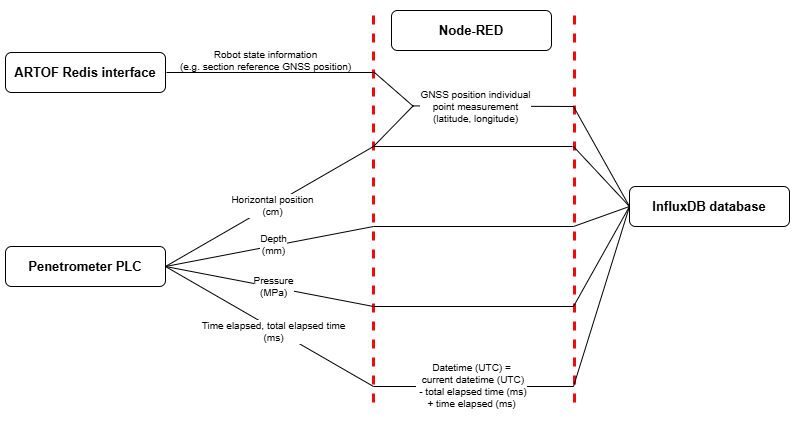

Supplement: Supplementary file 1 [file sensors-25-01919-s001.zip › FigureS1.png]

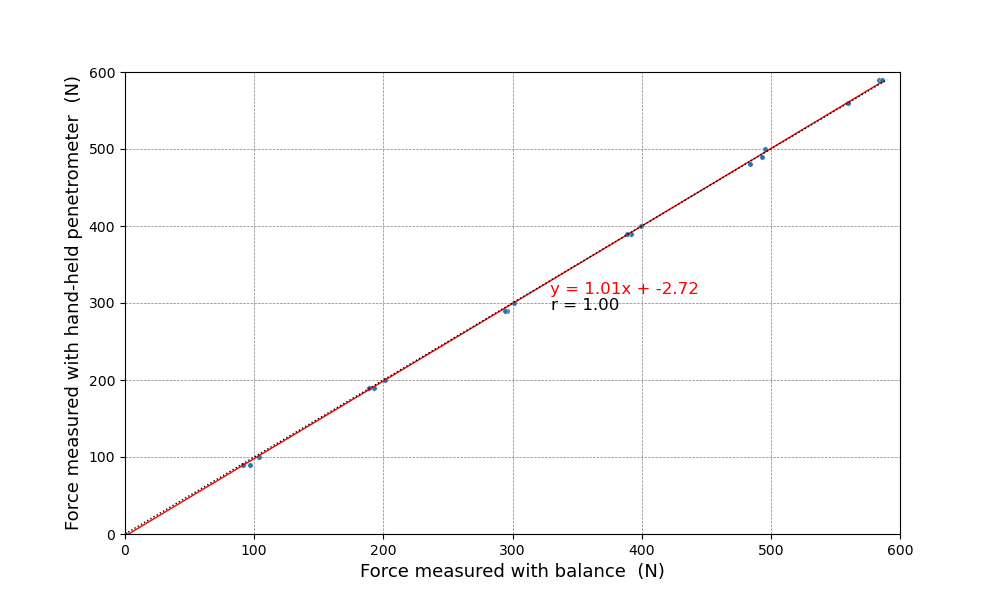

Supplement: Supplementary file 1 [file sensors-25-01919-s001.zip › FigureS2.png]

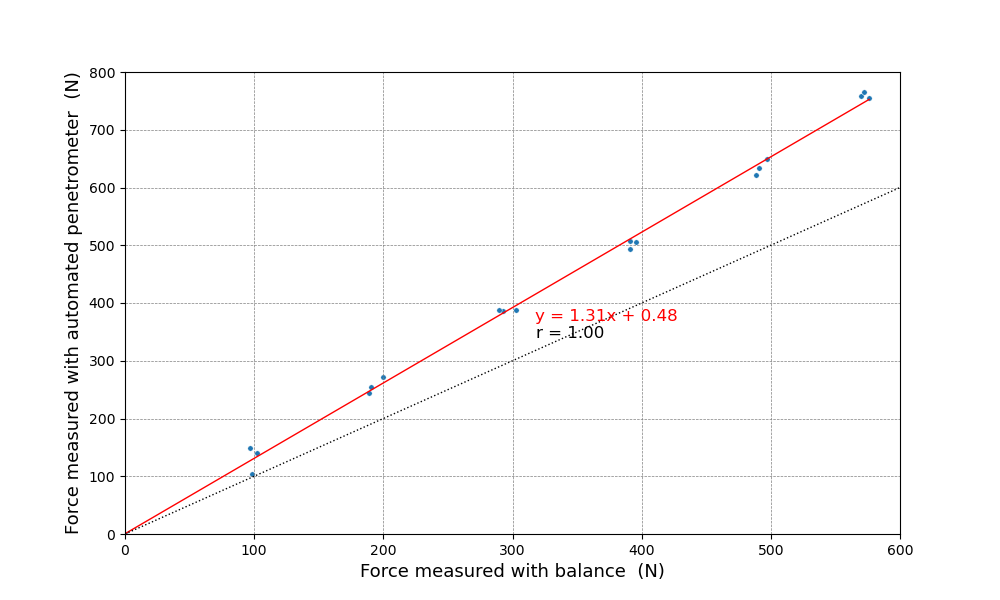

Supplement: Supplementary file 1 [file sensors-25-01919-s001.zip › FigureS3.png]
